# High prevalence of *bla_VEB_*, *bla_GES_* and *bla_PER_* genes in beta-lactam resistant clinical isolates of *Pseudomonas aeruginosa*

**DOI:** 10.3934/microbiol.2022013

**Published:** 2022-04-25

**Authors:** Saboura Haghighi, Hamid Reza Goli

**Affiliations:** 1 Sana Institute of Higher Education, Sari, Mazandaran, Iran; 2 Department of Medical Microbiology and Virology, Faculty of Medicine, Mazandaran University of Medical Sciences, Sari, Mazandaran, Iran

**Keywords:** *Pseudomonas aeruginosa*, *bla_VEB_*, *bla_GES_*, *bla_PER_*, beta-lactam resistance

## Abstract

The increased prevalence of β-lactamase is one of the main factors in resistance to β-lactams in *Pseudomonas aeruginosa*. This study aimed to investigate the prevalence of *bla_VEB_*, *bla_PER_*, and *bla_GES_* genes in β-lactam-resistant *P. aeruginosa*. We collected 100 non-duplicated clinical isolates of *P. aeruginosa* and identified them by standard tests. Using disk agar diffusion test, we detected the β-lactam-resistant isolates and extracted the DNAs of the isolates by alkaline lysis method. Then, the prevalence of *bla_VEB_*, *bla_PER_* and *bla_GES_* genes were detected by PCR method. The results were assessed by SPSS 21 software and Chi-square test. Out of 100 isolates, 43% were detected as resistant against at least one of the beta-lactams tested. Piperacillin-tazobactam was the most effective antibiotic, while 39% and 37% of the isolates were resistant to aztreonam and meropenem, respectively. A significant relationship was observed between the resistance to tested antibiotics and the presence of *bla_VEB_*, *bla_GES_*, and *bla_PER_* genes. Among 43 isolates that were resistant to at least one of the tested β-lactams, 93.02%, 83.72%, and 81.39% of them carried *bla_VEB_*, *bla_GES_*, and *bla_PER_* genes, respectively. According to this study and due to high prevalence of β-lactam resistance genes, it is better to check the level of antibiotic resistance and resistance genes for better management of patients with infection caused by this bacterium. Also, high prevalence of class A β-lactamases indicates the significant role of these enzymes in emerging resistance to beta-lactams.

## 1. Introduction

Among all the bacteria that cause nosocomial infections, gram-negative bacilli are of particular importance, while *Pseudomonas aeruginosa* has the highest priority among these bacteria [1]. *P. aeruginosa* is a non-fermenter gram-negative bacillus commonly found in hospitals and plays an important role in the development of different acute and chronic nosocomial infections [1],[2]. The large genome of *P. aeruginosa* (5.5–7 Mbp) enables high metabolic and environmental changes adaptability in this organism [3]. Multiple virulence factors, simple nutritional requirements, and high antimicrobials resistance rate, make this organism as an extremely dangerous pathogen [4],[5]. Treating infections caused by this bacterium has become a major challenge due to the organism's ability to develop high-level antibiotic resistance, as the World Health Organization (WHO) recently introduced the carbapenem-resistant *P. aeruginosa* as one of the bacteria needing the new drugs [6]. *P. aeruginosa* can be resistant to a variety of antibiotics, including aminoglycosides, quinolones, and β-lactams, by different inherently or acquired resistance mechanisms [7]. The β-lactam antibiotics, which contain β-lactam rings in their molecular structure, stop the biosynthesis of bacterial cell walls by targeting penicillin-binding proteins [7]. The annual cost of these antibiotics is approximately $ 15 billion and accounts for 65% of the total antibiotic market, while antibiotic resistance against these classes is increasing [7],[8]. The previous studies conducted in Asian countries in 2019 and 2020 showed that almost 12–39% of Iranian *P. aeruginosa* isolates and 26–60% of Gram-negative bacilli collected from Saudi Arabia were resistant to different β-lactams [2],[9]. Also, Nasser et al. exhibited that 3–100% of *P. aeruginosa* isolates collected in the Arab region from 2011 to 2018 were resistant against various β-lactams [10].

Resistance to these drugs can occur by a variety of mechanisms but is mainly due to the production of β-lactamases [9],[11]. As a causative agent of nosocomial infections, β-lactamase producing *P. aeruginosa* is one of the most harmful organisms to humans [10]. Among four classes of β-lactamases [12], class A beta-lactamase genes can be on plasmid, integron, or chromosome, and have the highest distribution among gram-negative bacteria [13]. Among these enzymes, some beta-lactamases such as Pseudomonas Extended Resistant (PER), Guiana extended-spectrum β-lactamases (GES), and Vietnam Extended Spectrum Beta-lactamase (VEB), which are part of extended-spectrum β-lactamases (ESBLs), are highly prevalent and significant in clinical isolates of *P. aeruginosa* [14]. The PER β-lactamase hydrolyzes most penicillins and cephalosporins and is mostly found in isolates from Turkey and Mediterranean countries [15],[16]. The VEB enzyme also promotes resistance to ceftazidime, aztreonam, and cefepime and is well inhibited by clavulanate and avibactam [17]. The GES family, as the carbapenemases, are more common in *P. aeruginosa* isolates and inhibited by clavulanate, tazobactam, avibactam, relebactam, and vaborbactam, however, the GES-1 hydrolyzes ceftazidime better than cefotaxime [18]. The genes encoding these three enzymes are usually carried by transposons and can move between Gram-negative bacteria [19]. Class A enzymes play a significant role in emerging resistance to β-lactams. Many studies have been published on these enzymes and their role in gram-negative bacilli. However, there is an urgent need for further studies on how these enzymes are resistant to clinically important β-lactams. Therefore, due to the great importance of the presence of PER, VEB, and GES β-lactamase encoding genes in the development of resistance to β-lactams, we aimed to evaluate the prevalence of these genes in clinical isolates of *P. aeruginosa* in this region of Iran.

## 2. Materials and methods

### 2.1. Ethical approval statements

Although the clinical samples were transferred to the Department of Medical Microbiology from the laboratories of the hospitals affiliated with the Mazandaran University of Medical Sciences, a written informed permission form was provided by the patients or a close relative, and organizing evidence of each sample was reserved secret. Also, this study was directed in agreement with the Declaration of Helsinki.

### 2.2. Bacterial isolation and identification

We collected 100 non-repetitive *P. aeruginosa* isolates from different clinical specimens. The specimens were included urine, sputum, eye secretion, catheter, wound, stool, and blood. These samples were collected from patients hospitalized in 5 therapeutic and educational hospitals affiliated with Mazandaran University of Medical Sciences, Sari, Iran, from 2018 to 2019. The isolates were identified by the standard microbiological and biochemical tests such as gram staining, pigment production on Müller-Hinton agar medium (Merck, Germany), non-fermentation reaction, and growth at 42 °C in triple sugar iron (TSI) agar (Merck), motility, citrate utilization, oxidase test, oxidation/fermentation (OF) test, and colony odor [20]. Then, the bacteria were frozen at −20 °C in trypticase soy broth (TSB) (Merck) containing 10% glycerol until use.

### 2.3. Antimicrobial susceptibility testing

We used the disk agar diffusion method to determine the antibiotic resistance pattern of the *P. aeruginosa* clinical isolates according to the guidelines of the Clinical and Laboratory Standards Institute (CLSI) [21]. In this study, 8 β-lactam antibiotics along with a β-lactam/ β-lactamase inhibitor combination including piperacillin (10 µg), piperacillin-tazobactam (10–110 µg), aztreonam (30 µg), cefepime (30 µg), ceftazidime (30 µg), imipenem (10 µg), meropenem (10 µg) and doripenem (10 µg) (MAST, UK) were used. After 18 h incubation of the cultures at 37 °C, we measured the zone of no bacterial growth around the antibiotic disks by ruler and reported the results as resistant, intermediate resistant, or susceptible. *P. aeruginosa* ATCC 27853 was chosen as a control strain in antimicrobial susceptibility testing.

### 2.4. DNA extraction from the bacterial isolates

We used the alkaline lysis method by adding sodium dodecyl sulfate (SDS) and NaOH for the extraction of the genomic DNAs of *P. aeruginosa* clinical isolates [22]. Briefly, we prepare an extraction solution by dissolving 0.5 g of SDS (Sigma, Germany) and 0.4 g of NaOH (Sigma) in 200 µL of sterile distilled water. Next, 4–6 colonies of pure bacteria were dissolved in 20 µL of this solution in a microtube. Then, the microtube was placed at 95 °C for 10 min and centrifuged for 3 min at 13000 g. Finally, 180 µL of sterile distilled water was added to the microtube and the supernatant was used as the extracted DNA. The OD (Optical Density) of the DNAs was measured using a NanoDrop (ND1000, USA). Also, the extracted DNAs were electrophoresed on 1.5% agarose gel (Wizbiosolutions, South Korea). Lastly, the extracted DNAs were stored in a freezer at −20 °C.

### 2.5. Detection of resistance genes using PCR

We used the specific primers (Metabion, Germany) including *veb*-forward-5′-CGACTTCCATTTCCCGATGC-3′ and *veb*-revers-5′-GGACTCTGCAACAAATACGC-3′ for detection of *bla_VEB_* gene with a product size of 642 bp [23], *per*-forward-5′-ATGAATGTCATTATAAAAGC-3′ and *per*-revers-5′-TTAATTTGGGCTTAGGG-3′ for detection of *bla_PER_* gene with a product size of 933 bp [24], and *ges*-forward-5′-GTTTTGCAATGTGCTCAACG-3′ and *ges*-revers-5′-TGCCATAGCAATAGGCGTAG-3′ for detection of *bla_GES_* gene with a product size of 387 bp [25] by PCR test. The PCR reaction was carried out in a final volume of 15 µL containing 7.5 µL of PCR Master Mix (Ampliqon, Denmark), 5 pmol of each primer, 5.5 µL of distilled water, and 300 ng of DNA. The initial denaturation step was done at 94 °C for 5 min. Then, 34 cycles of the amplification were performed as follows: Denaturation at 94 °C for 45 sec, annealing step for 20 sec at 63 °C for *bla_VEB_*, 55 °C for *bla_GES_*, and 52 °C for *bla_PER_*, and extension at 72 °C for 25 sec. A final extension step was used at 72 °C for 10 min. Then, the PCR product was electrophoresed on 1% agarose gel (Wizbiosolutiotions) with 1.5 µL of Safe Stain (SinaClon, Iran).

### 2.6. Statistical analysis

The data was introduced into SPSS software version 22, and the desired results were statistically analyzed using Pearson's Chi-Square test. *P-values* < 0.05 was considered statistically significant.

## 3. Results

### 3.1. Clinical data and isolation of the bacteria

We collected 100 non-duplicated *P. aeruginosa* isolates from 100 non-repeated inpatients in 5 educational and treatment hospitals including Imam Khomeini Hospital (40 isolates), Razi Hospital (22 isolates), Bu-Ali Sina Hospital (17 isolates), Zare Hospital (11 isolates) and Fatemeh Al-Zahra Hospital (10 isolates) affiliated to Mazandaran University of Medical Sciences, Sari, Iran. Out of 100 isolates, 60 of them were gotten from men. The mean age of women was 47.85 and the mean age of men was 44.76. The bacterial isolates were collected from respiratory samples (37 isolates), urine (26 isolates), wounds (20 isolates), blood (5 isolates), ocular discharge (2 isolates), stool (2 isolates), and catheters (8 isolates). Moreover, among 100 clinical isolates of this study, 53, 13, 6, 6, 5, 5, 4, 3, 2, 2, and 1 isolates were collected from intensive care units (ICUs), emergency, burn ward, operating room and surgery, cardiac care units (CCUs), pediatric ward, internal, men, women, neurology, and oncology wards, respectively.

### 3.2. Antibiotic resistance pattern of the isolates

**Figure 1.**
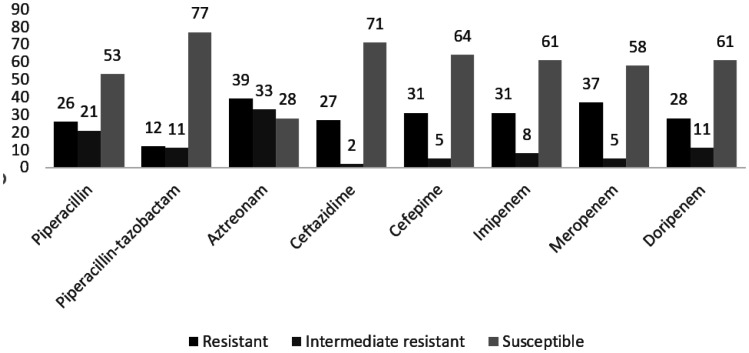
Antimicrobial resistance pattern of 100 *P. aeruginosa* clinical isolates in this study.

The antibiotic resistance pattern of *P. aeruginosa* clinical isolates against the 8 antibiotics tested in this study is shown in Figure 1. However, aztreonam was the least effective antibiotic due to a 39% resistance rate, while just 12% of the isolates were resistant against piperacillin-tazobactam. Moreover, 4% of the isolates were resistant to all tested antibiotics. Also, most intermediate resistance rate was shown against aztreonam, while just 2% of the isolates showed this phenotype against ceftazidime.

Also, Table 1 shows the antibiotic susceptibility pattern of the *P. aeruginosa* clinical isolates regarding the hospital wards in which the bacteria were isolated. According to this data, the highest resistance was related to the bacteria isolated from the burn ward. However, the resistance rates in surgery units and operating rooms were considerable.

**Table 1.** Number (%) of antibiotic resistant *P. aeruginosa* clinical isolates considering the hospital ward.

Hospital wards	ICU (n = 53)	Emergency (n = 13)	Burn (n = 6)	Surgery (n = 6)	CCU (n = 5)	Pediatric (n = 5)	Internal (n = 4)	Men (n = 3)	Women (n = 2)	Neurology (n = 2)	Oncology (n = 1)
Antibiotics											
Piperacillin	15 (28.3)	4 (30.76)	3 (50)	1 (16.66)	-	1 (20)	1 (25)	1 (33.33)	-	-	-
Piperacillin-tazobactam	7 (13.2)	2 (15.38)	1 (16.66)	1 (16.66)	-	-	-	1 (33.33)	-	-	-
Aztreonam	21 (39.62)	5 (38.46)	5 (83.33)	4 (66.66)	-	-	1 (25)	1 (33.33)	1 (50)	-	1 (100)
Ceftazidime	16 (30.18)	4 (30.76)	2 (33.33)	2 (33.33)	-	1 (20)	1 (25)	1 (33.33)	-	-	-
Cefepime	18 (33.96)	3 (23.07)	4 (66.66)	3 (50)	-	1 (20)	1 (25)	1 (33.33)	-	-	-
Imipenem	19 (35.84)	2 (15.38)	1 (16.66)	2 (33.33)	1 (20)	2 (40)	2 (50)	1 (33.33)	-	1 (50)	-
Meropenem	21 (39.62)	2 (15.38)	3 (50)	3 (50)	1 (20)	2 (40)	2 (50)	1 (33.33)	1 (50)	1 (50)	-
Doripenem	15 (28.3)	2 (15.38)	3 (50)	2 (33.33)	-	2 (40)	2 (50)	1 (33.33)	-	1 (50)	-

### 3.3. Identification of bla_VEB_, bla_GES_, and bla_PER_ genes by PCR

In the present study, 40%, 36%, and 35% of *P. aeruginosa* clinical isolates carried *bla_VEB_*, *bla_GES_*, and *bla_PER_* genes, respectively. Also, according to Table 2, which shows the number of β-lactam resistant isolates containing the studied genes, a significant relationship was observed between the presence of *bla_VEB_*, *bla_GES_*, and *bla_PER_* genes with resistance to the studied beta-lactams (P-*value* < 0.05). Among the 43 isolates that were resistant to at least one of the tested β-lactams, 40 (93.02%), 36 (83.72%), and 35 (81.39%) isolates carried the *bla_VEB_*, *bla_GES_*, and *bla_PER_* genes, respectively. Out of the 3 *bla_VEB_*-negative isolates, one was only susceptible to piperacillin-tazobactam and the other two isolates were only susceptible to ceftazidime. Among the 7 isolates without the *bla_GES_* gene, only one was resistant to piperacillin-tazobactam, while 2 isolates were intermediate resistant and 4 isolates were susceptible to this antibiotic. Isolates without the *bla_PER_* gene had the same situation. Although the presence of these genes was significantly associated with the development of resistance to the tested β-lactams, this association was slightly weaker for carbapenems, while resistance to doripenem was most strongly associated with the presence of these genes among carbapenems. However, among 43 β-lactam resistant isolates in this study, 34 (79.06%), 33 (76.74%), 31 (72.09%), and 30 (69.76%) isolates contained *bla_VEB_* + *bla_GES_*, *bla_VEB_* + *bla_PER_*, *bla_GES_* + *bla_PER_*, and *bla_VEB_ + bla_GES_ + bla_PER_* genes, respectively.

**Table 2.** Relationship between the presence of *bla_VEB_*, *bla_GES_*, and *bla_PER_* genes in clinical isolates of *P. aeruginosa* and resistance to beta-lactams.

Genes		Number (%) of isolates containing β-lactamase encoding genes					
		*bla_VEB_*		*bla_GES_*		*bla_PER_*	
Antibiotics and antibiotic resistance pattern (number)		positive	negative	positive	negative	positive	negative
Piperacillin	R (26)	24 (92.3)	2 (7.69)	23 (88.46)	3 (11.53)	25 (96.15)	1 (3.84)
	I (21)	12 (57.14)	9 (42.85)	11 (52.38)	10 (47.61)	9 (42.85)	12 (57.14)
	S (53)	4 (7.54)	49 (92.45)	2 (3.77)	51 (96.22)	1 (1.18)	52 (98.11)

Piperacillin-tazobactam	R (12)	11 (91.66)	1 (8.33)	11 (91.66)	1 (8.33)	11 (91.66)	1 (8.33)
	I (11)	10 (90.9)	1 (9.09)	9 (81.81)	2 (18.18)	9 (81.81)	2 (18.18)
	S (77)	19 (24.67)	58 (75.32)	16 (20.77)	61 (79.22)	15 (19.48)	62 (80.51)

Aztreonam	R (39)	36 (92.3)	3 (7.69)	33 (84.61)	6 (15.38)	32 (82.05)	7 (17.94)
	I (33)	4 (12.12)	29 (87.87)	3 (9.09)	30 (90.9)	3 (9.09)	30 (90.9)
	S (28)	-	28 (100)	-	28 (100)	-	28 (100)

Ceftazidime	R (27)	26 (96.29)	1 (3.7)	24 (88.88)	3 (11.11)	26 (96.29)	1 (3.7)
	I (2)	2 (100)	-	2 (100)	-	1 (50)	1 (50)
	S (71)	12 (16.9)	59 (83.09)	10 (14.08)	61 (85.91)	8 (11.26)	63 (88.73)

Cefepime	R (31)	28 (90.32)	3 (9.67)	28 (90.32)	3 (9.67)	29 (93.54)	2 (6.45)
	I (5)	5 (100)	-	4 (80)	1 (20)	3 (60)	2 (40)
	S (64)	7 (10.93)	57 (89.06)	4 (6.25)	60 (93.75)	3 (4.68)	61 (95.31)

Imipenem	R (31)	21 (67.74)	10 (32.25)	20 (64.51)	11 (35.48)	20 (64.51)	11 (35.48)
	I (8)	4 (50)	4 (50)	4 (50)	4 (50)	4 (50)	4 (50)
	S (61)	15 (24.59)	46 (75.4)	12 (19.67)	49 (80.32)	11 (18.03)	50 (81.96)

Meropenem	R (37)	27 (72.97)	10 (27.02)	25 (67.56)	12 (32.43)	26 (70.27)	11 (29.72)
	I (5)	3 (60)	2 (40)	3 (60)	2 (40)	3 (60)	2 (40)
	S (58)	10 (17.24)	48 (82.75)	8 (13.79)	50 (86.2)	6 (10.34)	52 (89.65)

Doripenem	R (28)	23 (82.14)	5 (17.85)	21 (75)	7 (25)	22 (78.57)	6 (22.14)
	I (11)	6 (54.54)	5 (45.45)	6 (54.54)	5 (45.45)	6 (54.54)	5 (45.45)
	S (61)	11 (18.03)	50 (81.96)	9 (14.75)	52 (85.24)	8 (13.11)	53 (86.88)

Abbreviations: R; resistant, I; intermediate resistant, S; susceptible

**Table 3.** Relationship between the presence of *bla_VEB_*, *bla_GES_*, and *bla_PER_* genes with the hospital ward related to sample collection.

Hospital wards	No. (%) of isolates carrying the relevant genes in the desired hospital wards					
	*bla_VEB_*		*bla_GES_*		*bla_PER_*	
	Positive	Negative	Positive	Negative	Positive	Negative
ICU (n = 53)	21 (39.62)	32 (60.37)	20 (37.73)	33 (62.26)	20 (37.73)	33 (62.26)
Emergency (n = 13)	5 (38.46)	8 (61.53)	5 (38.46)	8 (61.53)	4 (30.76)	9 (69.23)
Burn (n = 6)	4 (66.66)	2 (33.33)	5 (83.33)	1 (16.66)	5 (83.33)	1 (16.66)
Surgery (n = 6)	4 (66.66)	2 (33.33)	2 (33.33)	4 (66.66)	2 (33.33)	4 (66.66)
Pediatric (n = 5)	1 (20)	4 (80)	-	5 (100)	1 (20)	4 (80)
CCU (n = 5)	-	5 (100)	-	5 (100)	-	5 (100)
Internal (n = 4)	3 (75)	1 (25)	2 (50)	2 (50)	2 (50)	2 (50)
Men (n = 3)	1 (33.33)	2 (66.66)	1 (33.33)	2 (66.66)	1 (33.33)	2 (66.66)
Women (n = 2)	-	2 (100)	-	2 (100)	-	2 (100)
Neurology (n = 2)	-	2 (100)	-	2 (100)	-	2 (100)
Oncology (n = 1)	1 (100)	-	1 (100)	-	-	1 (100)

Moreover, Table 3 shows the relationship between the presence of the studied genes and the hospital wards of bacterial isolation. While most of the presence of resistance genes was observed in isolates collected from the burn section, no statistically significant relationship was observed in this regard. However, after assessing the relationship between the presence of genes and clinical sample type, we concluded that the presence of *bla_VEB_* and *bla_GES_* genes had a statistically significant relationship with the type of clinical samples and the highest presence of genes was observed in isolates collected from the wounds and catheters (Table 4).

**Table 4.** Relationship between the presence of *bla_VEB_*, *bla_GES_*, and *bla_PER_* genes in *P. aeruginosa* isolates and the type of clinical sample

Clinical samples	No. (%) of isolates carrying the relevant gene in the desired clinical samples					
	*bla_VEB_*		*bla_GES_*		*bla_PER_*	
	Positive	Negative	Positive	Negative	Positive	Negative
Urine (n = 26)	7 (26.92)	19 (73.03)	6 (23.07)	20 (76.92)	7 (26.92)	19 (73.03)
Respiratory (n = 37)	12 (32.43)	25 (67.56)	11 (29.72)	26 (70.27)	9 (24.32)	28 (75.67)
Wound (n = 20)	11 (55)	9 (45)	12 (60)	8 (40)	12 (60)	8 (40)
Catheter (n = 8)	7 (87.5)	1 (12.5)	5 (62.5)	3 (37.5)	4 (50)	4 (50)
Blood (n = 5)	2 (40)	3 (60)	2 (40)	3 (60)	2 (40)	3 (60)
Stool (n = 2)	-	2 (100)	-	2 (100)	-	2 (100)
Eye (n = 2)	1 (50)	1 (50)	-	2 (100)	1 (50)	1 (50)

## 4. Discussion

*Pseudomonas aeruginosa* is a gram-negative opportunistic pathogen and one of the leading causes of nosocomial infections in immunocompromised patients, including patients with a wide range of malignancies, cystic fibrosis, burns, and others [26]. Suitable drugs against infections caused by this bacterium are aminoglycosides, fluoroquinolones, selected β-lactams (e.g., ticarcillin, piperacillin, ceftazidime, aztreonam, and carbapenems), and a β-lactam/β-lactamase inhibitor compound [27]. Treating *P. aeruginosa* infections has become a major challenge due to the bacterium's ability to develop widespread antibiotic resistance [28]. One of the most prominent features of these bacteria is their resistance to clinically important antibiotics such as third-generation cephalosporins, imipenem, aztreonam, and other important beta-lactams [29].

Acquired resistance to beta-lactams is due to the production of β-lactamase enzymes such as broad-spectrum beta-lactamases (ESBLs), Metallo-β-lactamases (MBLs), and sometimes AmpC plasmid β-lactamases [28]. Beta-lactamases are classified as A, B, C, and D groups by Ambler [12] while sequencing on class A β-lactamase indicated the presence of subclasses A1 and A2 with 100% similarity and highly conserved [30]. The GES enzyme belonged to subgroup A1 of class A ESBLs, is detected in a wide range of countries, and is encoded mainly by plasmids or integrons [30]. These enzymes hydrolyze penicillins, most broad-spectrum cephalosporins, aztreonam, and sometimes carbapenems but not cephamycins, and are inhibited by clavulanate, tazobactam, or imipenem [31],[32]. However, 24 (61.53%), 28 (66.66%), and 27 (69.23%) isolates with resistant or intermediate resistant phenotype to imipenem, meropenem, and doripenem in our study carried the *bla_GES_* gene, respectively. This indicates that the presence of this gene may contribute in the development of resistance to carbapenems in our study (P-*value* = 0.000).

On the other hand, the enzyme *bla_GES_*_-11_ hydrolyzes cefotaxime, ceftazidime, and aztreonam more efficiently than *bla_GES_*-1 and is more susceptible to clavulanate and tazobactam [31]. Although 84.61% and 88.88% of the aztreonam and ceftazidime resistant isolates in our study carried the *bla_GES_* gene, 91.66% of the isolates that were resistant to piperacillin-tazobactam also carried this gene, indicating that the *bla_GES_*_-11_ enzyme may not have been involved in our study. In a study conducted by an Iranian group on 120 burn *P. aeruginosa* isolates, 41 isolates were identified as ESBL-producer, while among them, 10 (24.4%) isolates were carrying the *bla_GES-1_* gene [33], while in 2009, a similar study was performed on MDR *P. aeruginosa* isolates in Tehran and no isolates carried this gene [34]. Increased prevalence of this gene in recent years in Iran and other countries can be a serious warning about the increasing resistance of clinical *P. aeruginosa* isolates to carbapenems in different parts of the world, while these antibiotics are the last line treatment option before colistin [35]. Another study on 17 *P. aeruginosa* isolates in a private hospital in Durban, South Africa reported that 13 isolates were ESBL positive, while 10 (58.82%) isolates carried the *bla_GES-2_* gene [36]. This gene hydrolyzes broad-spectrum cephalosporins and imipenem at low levels [36], while 96.29% and 67.74% of the ceftazidime and imipenem resistant isolates in our study carried the *bla_GES_* gene. This suggests that other resistance mechanisms may have been involved, or that the gene identified in our study was not *bla_GES-2_*. In another study conducted in Hamedan, 43/88 (48.88%) *P. aeruginosa* isolates were ESBL positive and 2 (2.2%) Isolates carried the *bla_GES_* gene [25].

On the other hand, phylogenetic analysis of β-lactamases has shown the presence of genes transmissible through plasmids and integrons such as *bla_VEB_* and *bla_PER_* in *P. aeruginosa* [30]. These enzymes are mainly hydrolyzing the cephalosporins, such as cephalothin, ceftazidime, and cefotaxime, as well as aztreonam and penicillins, and fall into class A2 of the Ambler classification [30]. PER-1 and PER-2 are the most common members of the PER family and are less inhibited by avibactam than other class A β-lactamases [18]. However, 93.54% and 64.51% of the isolates resistant to cefepime and imipenem in our study carried the *bla_PER_* gene, respectively, while 70.27% and 78.57% of the meropenem and doripenem resistant isolates were carrying this gene, respectively.

VEB-1 enzyme is an effective agent in resistance to ceftazidime, cefotaxime, cefepime, and aztreonam but not imipenem, and can be inhibited by clavulanate [17]. In the present study, 92.3%, 96.29%, and 90.32% of the isolates resistant to aztreonam, ceftazidime, and cefepime carried this gene, indicating the important role of the VEB enzyme in resistance to these antibiotics. Importantly, 67.74%, 72.97%, and 82.14% of the isolates resistant to imipenem, meropenem, and doripenem in our study also carried this gene, but according to the above reference data, other mechanisms or β-lactamase enzymes, including Metallo-β-lactamase, were involved in the development of these resistances. On the other hand, due to the fact that VEB and PER enzymes are inhibited by tazobactam [37], 91.66% of piperacillin-tazobactam resistant isolates in our study also carried this gene, which indicates that the enzyme is not inhibited by tazobactam. Croughs et al., from the Netherlands, reported that out of 1528 *P. aeruginosa* isolates, 113 were ceftazidime resistant and only 4 (5.3%) isolates had ESBLs, and 2 of these isolates carried the *bla_VEB-2_* gene [38]. Laudy et al., from Poland, reported that out of 900 *P. aeruginosa* isolates, 99 (11%) cases were ESBL positive, while 69 isolates had *bla_VEB-9_* gene and 14 isolates carrying *bla_GES_* genes including *bla_GES-1_* (n = 6), *bla_GES-5_* (n = 1), *bla_GES-13_* (n = 5), and *bla_GES-15_* (n = 2) [39]. In a study conducted in Greece, 29% of the isolates were resistant to ceftazidime, while 1.7% were ESBL positive, and among them, 5 isolates carried the *bla_PER-1_* gene [16]. Also, in a study conducted in 2015 on 200 camel meat samples in Egypt, 45 isolates of *P. aeruginosa* were obtained, while 21 (46.66%) isolates were identified as ESBL positive and 28.5% of these isolates carried the *bla_PER-1_* gene [24]. This result indicates the importance of transmission of this gene through mobile genetic elements, while the *bla_PER-1_* gene was the most common (55%) class A ESBL gene after the *TEM* gene (87.5%) in another study on clinical isolates of *Acinetobacter baumannii* in Egypt [40]. Also, 27.5% of their isolates carried the *bla_GES_* gene, but the *bla_VEB_* gene was not observed in any of the isolates [40]. Also, in an Egyptian study conducted by Zafer et al. in 2014, 7.4% of the *P. aeruginosa* isolates were ESBL positive, while 10.4% of them carried the *bla_VEB-1_* gene, and 60.6%, 45.1%, and 25.4% of their isolates were resistant to ceftazidime, aztreonam, and piperacillin-tazobactam, respectively [35].

Moreover, other Iranian studies conducted in 2010 on burn isolates of *P. aeruginosa* reported that 100% and 31.34% of the isolates contained *bla_VEB-1_* gene, while the *bla_PER-1_* gene was identified in 68.3% and 49.25% of ESBL positive isolates, respectively [33],[41]. Moreover, another study in Hamedan, Iran, showed that their 16 (26.6%) and 9 (15%) ESBL positive isolates contained the *bla_PER-1_* and *bla_VEB-1_* genes [26], while in a study conducted in Ahvaz, the *bla_PER-1_* and *bla_VEB-1_* genes were identified in 2 (6.6%) and 4 (13.3%) ESBL positive isolates, respectively [29].

## 5. Conclusions

The emergence of antibiotic-resistant strains of *Pseudomonas aeruginosa* has posed a major challenge in the treatment of infections caused by this bacterium, and resistance to β-lactams as one of the common therapeutic families has also led to treatment deficiencies. On the other hand, the presence of genes encoding ESBLs will increase the rate of problems in the treatment of infections caused by this bacterium. Class A β-lactamases are important in developing resistance to β-lactams. Due to the high ability of the genes encoding these enzymes to move between different organisms through integrons and plasmids, if the rapid spread of these genes is not prevented, none of the gram-negative bacteria will soon respond to any of the β-lactams. This becomes even more important when it causes widespread resistance to carbapenems. Among the most important enzymes in this group of β-lactamases are GES, VEB, and PER enzymes, which over time have increased their prevalence in Iran. Therefore, it is necessary to study the status of antibiotic resistance of *Pseudomonas aeruginosa* clinical isolates and identify the genes involved in the development of these resistances. However, the limitation of this research was the lack of sequencing of the *bla_VEB_*, *bla_PER_*, and *bla_GES_* genes which hampers the ability to connect back to the AST phenotypes.
